# The interplay between copper metabolism and microbes: in perspective of host copper-dependent ATPases ATP7A/B

**DOI:** 10.3389/fcimb.2023.1267931

**Published:** 2023-11-30

**Authors:** Yixuan Zhou, Leiliang Zhang

**Affiliations:** ^1^ Department of Clinical Laboratory Medicine, The First Affiliated Hospital of Shandong First Medical University & Shandong Provincial Qianfoshan Hospital, Jinan, China; ^2^ Department of Pathogen Biology, School of Clinical and Basic Medical Sciences, Shandong First Medical University & Shandong Academy of Medical Sciences, Jinan, China; ^3^ Medical Science and Technology Innovation Center, Shandong First Medical University & Shandong Academy of Medical Sciences, Jinan, China

**Keywords:** copper metabolism, microbes, ATP7A, ATP7B, membrane trafficking

## Abstract

Copper, a vital element in various physiological processes, is transported from the gastrointestinal tract to tissues and cells through diverse copper transporters. Among these transporters, ATP7A and ATP7B play significant roles in regulating systemic copper metabolism and exhibit precise regulation in their intracellular trafficking. These transporters undergo dynamic shuttling between the trans-Golgi network (TGN) and the plasma membrane via the endocytic recycling mechanism, which involves the retromer and other associated factors. Interestingly, the antimicrobial attribute of copper implies a potential connection between microbial infection and copper metabolism. Several microbes, including *Salmonella enterica*, *Cryptococcus*, Influenza A virus (IAV) and Zika virus (ZIKV) have been observed to impact the regulatory mechanisms of ATP7A/B, either directly or indirectly, as a means of survival. This review summarizes the key features and trafficking mechanisms of the copper transporters ATP7A/B, and examines the intricate interplay between microbes and copper metabolism. Ultimately, it highlights how microbes can perturb copper homeostasis through interactions with host factors, offering valuable insights into the mechanistic aspects of host-microbe interactions.

## 1 Introduction

Copper is a critical micronutrient involved in numerous physiological processes. Through redox reaction, it transfers electrons between cuprous (Cu^+^) and cupric (Cu^2+^) states, and this ability makes it an essential cofactor in redox enzymes. Those enzymes play vital roles in respiration (cytochrome c oxidase), peptide processing (peptidylglycine α-amidating mono-oxygenase), and protection of oxidative stress (superoxide dismutase SOD). (Medeiros and Jennings, 2002; Steiger et al., 2010) However, deficiencies or imbalances in copper metabolism can disrupt normal physiological functions, resulting in a spectrum of diseases such as Menkes disease, Wilson disease, anemia, and neurodegenerative disorders. (Chen et al., 2020) Additionally, impaired copper metabolism may cause cellular damage directly or indirectly. (Dupont et al., 2011) In order to make use of this essential but toxic substance, specific proteins are responsible for precisely regulating the systematic intake, distribution, and efflux of copper. After being reduced by reductases, cuprous ions (Cu^+^) are incorporated into cells by the copper transporter 1 (Ctr1) and subsequently assigned to different utilization pathways: cytosolic, mitochondrial, and Golgi routes. (Festa and Thiele, 2011).

**Figure 1:**
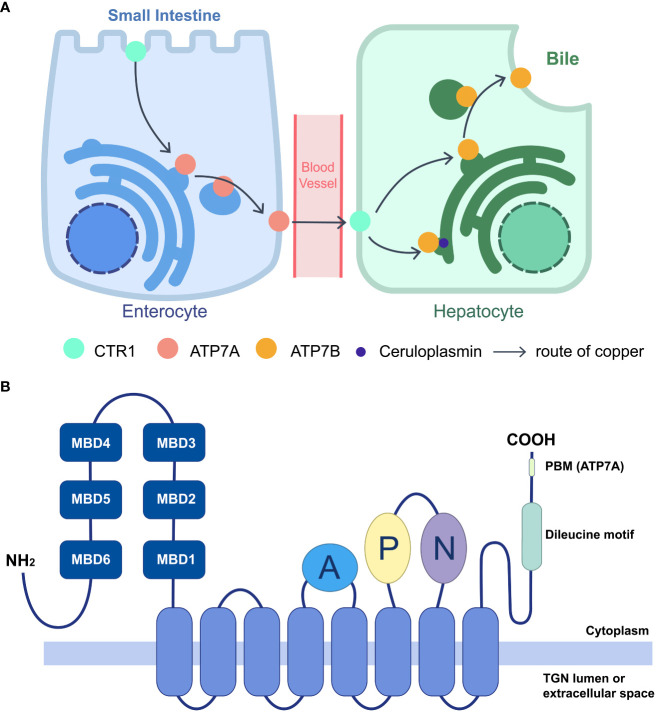
Function of ATP7A/B in systematic copper distribution and their domain structure. **(A)** Copper homeostasis is maintained largely by importer CTR1 and exporter ATP7A/B. Cu is absorbed in small intestine by enterocytes, through the apical CTR1, and the efflux is mediated by ATP7A, transporting Cu into blood vessels. After intake by CTR1 on hepatocytes, Cu is loaded on cuproproteins, including ceruloplasmin at TGN, or secreted to bile when redundant copper accumulating, which are both mediated by ATP7B. The lack of ATP7A/B would lead to different kinds of copper deficiency. **(B)** Schematic diagram of the membrane topology and main domains of the P1B-type ATPase, ATP7A/B. ATP7A/B contains an eight-helices transmembrane domain (marked blue) to form a pore, connecting with three cytoplasmic domains including A (marked purple), P (marked lemon), N (marked green). The N termini lies six MBDs (marked dark blue) and C termini contains a dileucine motif (marked green). For ATP7A, a class I PBM is located on the C terminus (DTAL).

Cu^+^ is transported to P-type ATPases, including ATPase copper-transporting α (ATP7A) and ATPase copper-transporting β (ATP7B), by the copper chaperone antioxidant protein 1 (Atox1). (Festa and Thiele, 2011) ATP7A and ATP7B are copper-dependent transporters that play essential roles in copper intake and distribution. They were initially identified in Menkes disease and Wilson disease, two disorders characterized by copper dysmetabolism. (Tanzi et al., 1993; Vulpe et al., 1993) ATP7A is widely expressed in most tissues except the liver, the primary organ of copper distribution. ATP7A transports copper ions from the small intestine to intracellular fluid in enterocytes. In contrast, ATP7B is involved in copper exportation. Excess copper is secreted from hepatocytes into the bile and eliminated from the body (**Figure 1A**). (Chen et al., 2020) Therefore, systemic dysfunction of ATP7A/B can cause severe copper deficiency or overload. However, partial abnormalities can result in specific symptoms, such as motor neuron-specific deficiency leading to muscle atrophy, progressive deterioration in motion, and denervation of neuromuscular junctions. (Hodgkinson et al., 2015).

Since the 2000s, evidence has accumulated to reveal a connection between copper metabolism and microbial infection. Research on bacteria like *Salmonella enteric (S. enteric), serovar Typhimurium* (*S. Typhimurium*), *Mycobacterium tuberculosis* (*M. tuberculosis*), and *Candida albicans* (*C. albicans*) has suggested that copper-deficient conditions enhance mortality rates and enable bacteria to survive better. (Newberne et al., 1968; Weissman et al., 2000; Ward et al., 2010) Viruses, such as Influenza A virus (IAV) and Zika virus (ZIKV), interfere with the host copper metabolism, which then generates reactive oxygen species (ROS), leads to oxidative stress in host cells, and possibly causes cellular autophagy. (Jung et al., 2018; Puig-Pijuan et al., 2022) Copper, in turn, catalyzes antimicrobial responses by damaging the protein functions of bacteria and viruses. (Dupont et al., 2011; Fujimori et al., 2012).

The world is currently facing challenges in preventing and controlling infectious diseases, including the emergence of new pathogens such as severe acute respiratory syndrome coronavirus 2 (SARS-CoV-2), re-emerging pathogens like invasive nontyphoidal Salmonella causing public health concerns, and recent outbreaks of mpox. To tackle these challenges, it is crucial to develop effective strategies for the prevention and control of infectious diseases. In this context, exploring the relationship between copper metabolism and microbial infections may provide novel insights into how the human body can effectively combat microbial invasions. This review summarizes the functions and features of copper-dependent ATPases and sheds light on the interplay between microbes and copper. We specially focused on the interaction between microorganisms and ATP7A/B, to uncover potential links paving the way for further research.

## 2 Features and trafficking mechanisms of copper-dependent ATPases

ATP7A and ATP7B are copper-dependent and copper-transporting proteins that employ adenosine 5′-triphosphate (ATP) to pump copper ions. They share the conserved structure with other P-type ATPases and both transport ions from intracellular compartments with low concentrations to extracellular fluid with high concentrations. (Dyla et al., 2020) The pumps consist primarily of three parts: a transmembrane domain, which forms the pore through eight membrane-spanning helices; three cytoplasmic ATP hydrolytic domains, which are an actuator (A) domain, a phosphorylation (P) domain, and a nucleotidebinding (N) domain; and an amino terminus comprising six consecutive metal-binding domains (MBDs) (**Figure 1B**). (Sazinsky et al., 2006; Dyla et al., 2020) On each MBD, a highly conserved copper-binding motif (GM(T/H)CxSCxxxIE) is found, which is broader than the traditional specific copper-specific motif (CxxC). (Yu et al., 2017) Various cuproproteins bind copper through the Atox1/ATP7B/Golgi pathway, including ceruloplasmin, the main copper-carrying protein in the blood, while ATP7A delivers copper to the Golgi apparatus for cuproenzyme metalation, such as SOD. (Chen et al., 2020).

**Figure 2:**
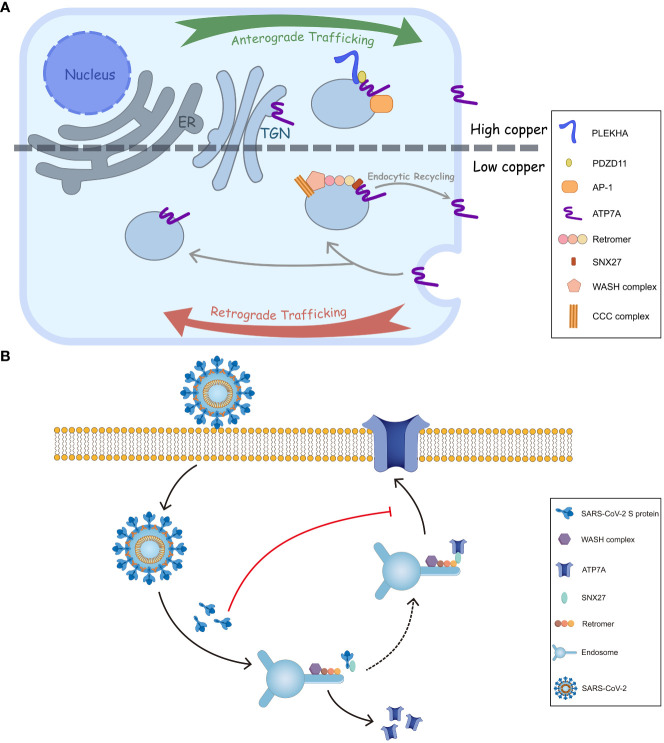
Intracellular trafficking of ATP7A and a proposed model of inhibition of ATP7A trafficking by SARS-CoV-2 S. **(A)** In response to elevated copper levels, ATP7A undergoes relocation from TGN to the plasma membrane. This movement is facilitated by the interactions of ATP7A with AP-1 and PDZD11-PLEKHA.When overloaded copper is secreted, the ATP7A pump is wrapped in endosome. The endosome is then sorted, allowing it to either return back to TGN or be retrieved and transported back to the plasma membrane through SNX27-retromer sorting. **(B)** The endocytic recycling of ATP7A is facilitated by the interaction between SNX27-Vps26A and ATP7A. However, SARS-CoV-2 S protein could inhibit the targeting of ATP7A to the plasma membrane by binding with SNX27 through its PDZ binding sequence. This binding event may interfere with the normal recycling of ATP7A, ultimately leading to a decrease of ATP7A in cell surface.

The trafficking of ATP7A/B largely relies on copper concentration. The ATPases, located on the trans-Golgi network (TGN) at basal copper levels, are stimulated by elevated copper, followed which they accumulate at cell periphery to expel excess Cu+ (**Figure 2A**). The phosphorylation level of the ATPases, corresponding to copper concentration, influences their cellular distribution. It was commonly acknowledged that hyperphosphorylation of ATP7A/B triggers their trafficking from TGN to the cell membrane, and dephosphorylation contributes to their retrieval, (Vanderwerf et al., 2001; Voskoboinik et al., 2003) where the formation of phosphorylated catalytic intermediates is necessary. (Petris et al., 2002) Phosphorylation sites have been identified at both the C- and N-termini, and, especially, those on the C-terminus are essential for intracellular transport of ATP7A/B. (Petris et al., 2002; Pilankatta et al., 2009; Braiterman et al., 2015) MBDs also play critical roles in copper transport and the trafficking process of ATP7A/B. In ATP7B, MBD2 first receives copper from Atox1, and two functional groups, MBD1-3 and MBD5-6, have been identified by nanobodies binding and nuclear magnetic resonance (NMR) relaxation. (Huang et al., 2014) Moreover, MBD4-6 are also indispensable because blocking MBD4-6 from copper binding results in decreased copper transport, which is consistent with the results of electron paramagnetic resonance (EPR) spectroscopy. (Shanmugavel and Wittung-Stafshede, 2019; Zaccak et al., 2020) Furthermore, metallothioneins (MTs), especially MT-I/II, are involved in ATP7A/B expression and trafficking. As a copper-binding protein, MTs enhance copper tolerance. The loss of both MTs and ATP7A, especially ATP7A, results in cell death because of copper accumulation; however, ATP7B expression is enhanced at the same time. The knockout of MTs increases ATP7A trafficking from the Golgi to the cell periphery because more copper is available for an elevated copper level. (Gudekar et al., 2020).

The membrane trafficking of ATP7A has also been explored. (Pascale et al., 2003; Holloway et al., 2007) The retromer, together with the Wiskott-Aldrich syndrome protein and SCAR homologue (WASH) complex and COMMD/CCDC22/CCDC93 (CCC) complex, among others, prevents lysosomal degradation and promotes the retrograde trafficking of ATP7A. (Phillips-Krawczak et al., 2015) Additionally, Steinberg et al. identified sorting nexin 27 (SNX27) and the retromer as cargo interactors, contributing to the recycling pathway and retrieving ATP7A back to the plasma membrane (**Figure 2A**). (Steinberg et al., 2013) As for ATP7B, a direct interaction with Vps35, a core component of retromer, has been discovered. Furthermore, COMMD1 of CCC complex also facilitates the retromer-mediated transport of ATP7B by interacting with WASH complex.

Besides the retromer complex, there are other factors engaged in ATP7A/B transports. Adaptor protein (AP) 1 and 2 function in secretion and endosomal recycling of ATP7A/B mediated by clathrin-coated vesicles. (Robinson, 2004) The C-terminal dileucine motif ([DE]XXXL[LI]) of ATP7A interacts with AP-1 and AP-2. It serves as a vital sorting signal for the proper localization of ATP7A (**Figure 1B**), and mutation on its C termini (P1386S) would disrupt the typical trafficking pattern. (Yi and Kaler, 2015) Like ATP7A, ATP7B interacts with AP-1 to complete its anterograde trafficking. (Jain et al., 2015) Sluysmans et al. proposed that PDZ domain-containing protein 11 (PDZD11), an interactor of the C-terminal 15 amino acids of ATP7A, and pleckstrin homology domain-containing family A (PLEKHA) members form a WW-PLEKHA-PDZD11 complex at high copper levels to mediate the anterograde trafficking of ATP7A. (Sluysmans et al., 2021) However, the thorough pathway of ATP7A/B trafficking has not been worked out, especially for ATP7B, and further research is needed to elucidate more detailed mechanisms.

## 3 Function of copper in antimicrobial responses

Copper has been used as a disinfector and medicine since ancient times for its antimicrobial feature. Moreover, copper-related treatments, such as compounds and nanoparticles, offer potential therapeutic alternatives which could replace traditional antibiotics. (Kaur et al., 2023) As previously mentioned that copper exists and cycles between reduced(Cu^+^) and oxidized(Cu^2+^) forms, it is required by the development of immune function. Copper status strongly influences several aspects of neutrophils and monocytes, (Maggini et al., 2007) and CTR1 and ATP7A/B, three essential copper transporters to copper level regulation, pave the way for copper toxicity for antimicrobial response. Correspondingly, a high susceptibility to urinary tract infections and septicemia is reported in cases of Menkes disease, which further proved the relation between copper and immunity. (Kreuder et al., 1993; Yoganathan et al., 2017) On the other side, copper-resistant ability is necessary for microbial survival. For example, *S. Typhimurium* largely depends on periplasmic copper-binding protein CueP and copper transporters as CopA and GolT to combat the toxicity of copper in macrophages. (Osman et al., 2010) That evidence revealed that there might be a link between copper transport and microbial infection.

Some mechanisms for copper toxicity to microbes are revealed. The copper-mediated antimicrobial effect can be explained in several aspects: (Dupont et al., 2011; Kaur et al., 2023) (1) copper ions bind to cell walls of bacteria, straightly impairing membrane proteins and conducting membrane perforation; (2) Fenton-like reaction mediated by copper and hydrogen peroxide generates ROS, causing lipids peroxidation and proteins oxidation, possibly impeding respiratory process of mitochondria (Li et al., 2019); (3) copper ions enjoy a high affinity to other metal ions, and replace them as zinc, inducing loss of protein function, or iron, destabilizing iron-sulfur (Fe-S) clusters of bacterial dehydratase enzymes; (Macomber and Imlay, 2009) other factors as osmotic pressure can be disturbed by copper ions, resulting in cell contents and nutrients leaking. Besides, copper accumulation also induces antiviral responses through degrading viral proteins, such as hemagglutinin (HA) and neuraminidase (NA) of the influenza virus. (Fujimori et al., 2012).

Moreover, copper functions in phagosomes of macrophages in response to microbial infection. Cu^+^ is imported by CTR1, accompanied by Atox1, and finally delivered to phagosomes through ATP7A. (Hodgkinson and Petris, 2012) When stimulated by cytokines as interferon-γ (INF-γ) and tumour necrosis factor-α (TNF-α) or exogenous toxicants as lipopolysaccharide (LPS), the expression of CTR1 and ATP7A would be upregulated to accumulate Cu^+^ in phagosomes, (Wagner et al., 2005; White et al., 2009) followed which ROS are generated. Under aerobic conditions, Cu^+^ catalyzes the Fenton and Haber–Weiss reactions, which generates hydroxyl radicals. (Halliwell and Gutteridge, 1990) By contrast, anaerobically, Cu^+^ binding with thiolates releases iron in Fe-S cluster, causing oxidative damage via iron-based Fenton reactions. (Keyer and Imlay, 1996).

## 4 Interplay between microbes and copper-dependent ATPases during infection

Several bacteria and viruses that interact with ATP7A/B have been extensively studied, but there is still a need to gain a better understanding of the relationship between microbes and copper metabolism mediated by these two proteins. *Enterobacteriaceae* members like *S. enterica*, *Escherichia coli (E. coli)*, and *Klebsiella pneumoniae* (*K. pneumoniae*) share common characteristics when it comes to combating copper attacks and interacting with host ATP7A. *M. tuberculosis* has been found to interact with host ATP7A through its prokaryotic ATPases. Fungi such as *Cryptococcus* can directly impact the expression levels of ATP7A. In terms of viruses, IAV and its association with ATP7A have been extensively studied and discussed. Other viruses, such as ZIKV, have garnered attention due to the potential link between copper deficiency and nervous system damage.

### 4.1 Salmonella enterica

*S. enterica* can be transmitted through animal-based foods and contaminated water or food sources. (Ferrari et al., 2019) This species is comprised of six subspecies and more than 2,500 serovars, including nontyphoidal *S. Typhimurium*, *S. Enteritidis* and typhoidal *S. Typhi*, of which the symptoms vary from gastroenteritis to a febrile systemic disease, finally to typhoid fever with sustained bacteremia. (Kurtz et al., 2017) Those serotypes are distinguished by different O (somatic) and H (flagellar) antigens. Once infecting the hosts, Salmonella attaches to M cells, antigen delivery cells located at the intestinal epithelium. Then, it is transmitted to macrophages, which are killed by Salmonella via type III secretion system.

As a leading cause of death among food-borne diseases, the survival of *S. enterica* in the phagosome of macrophages requires copper tolerance. This ability mainly depends on procaryotic copper transporters CopA and GolT, two P1B-type ATPases, pumping out Cu^+^ from cytosol to periplasm space in order to maintain the intracellular copper level for their survival under host copper attacks. (Hodgkinson and Petris, 2012) The total cellular copper accumulated when RAW 264.7 macrophages were infected with CopA and GolT genes deleted *S. Typhimurium*, using ICP-MS to measure washed extracts. Furthermore, the deletion of both *CopA* and *GolT* genes demonstrates a remarkable decrease in survival of *S. Typhimurium*.(Osman et al., 2010) CopA is encoded by copper-sensing transcriptional regulator (CueR), while GolT, uniquely expressed in *S. enterica*, belongs to GolST-B system. They are responsible for the delivery of Cu^+^ to periplasmic, followed by Cu^+^ oxidization via CueO, accompanied by CueP to activate superoxide dismutase SodCII and finally efflux. (Osman et al., 2013; Hyre et al., 2021) Additionally, *E. coli* shares a similar copper tolerance strategy with *S. enterica*, where CopA and CueO are encoded by CueR involving in cooper regulation. Interestingly, *K. pneumonia*, another closely related member of Enterobacteriaceae, also utilizes CopA in copper resistance, revealing a mechanism that defends the toxic host factors. (Bachman et al., 2015).

The brief mechanism regarding the interplay between *S. enterica* and host copper-regulating factors as ATPases is revealed. It had been previously elucidated that INF-γ and LPS could trigger the expression and trafficking to a phagolysosomal compartment in RAW264.7 macrophages. (White et al., 2009) Afterwards, Ladomersky et al. constructed *Atp7a^LysMcre^* mice, a myeloid-specific ATP7A knockout model, revealing that a higher percentage of *S. Typhimurium* recovered in *Atp7a^LysMcre^* macrophages within 2 hours after infection. (Ladomersky et al., 2017) They then detected the copper level in *Atp7a^LysMcre^* cells and measured the susceptibility of CopA and GolT knockout *S. Typhimurium* in the liver and spleen of *Atp7a^LysMcre^* mice and controls, concluding that bacterial ATPases CopA maintain phagosome copper levels and GolT is required for *S. Typhimurium* invasion, while ATP7A-dependent copper trafficking to phagosome controls the infection. Besides, *S. Typhimurium* triggered copper accumulation at ‘copper hot spots’ in bone-marrow-derived macrophages (BMM) via upregulating *Atp7a* gene 10 to 15 folds at 14 h post-infection. (Achard et al., 2012) However, the localization of copper hot spots is out of anticipation, where those spots illustrated no association with salmonella-containing vacuole, early endosomes and lysosomes but were isolated by a lipid membrane, suggesting that they might participate in a late-response antibacterial effect.

### 4.2 Mycobacterium tuberculosis

As the causative agent of tuberculosis, *M. tuberculosis* is responsible for more than 1 million deaths each year worldwide. It can be transmitted through the respiratory tracts, which then spreads via the bronchi or the lymphatics, causing tuberculosis diseases within 1 to 2 years. The typical symptoms are inflammation, mainly in the lung, leading to caseous pneumonia and fibrocaseous disease. (Mashabela et al., 2019) To survive in alveolar macrophages, its P1B-type ATPase CtpV plays an essential role in exporting copper through the inner membrane. Deleting the *ctpV* gene illustrates a lower rate of lung damage and killing in mice than wild-type bacteria. (Ward et al., 2010) In order to control the infection of *M. tuberculosis*, host macrophages utilize ATP7A to pump copper into phagosomes, conducting a copper nutritional immunity. CtpV and ctpB, another P1B-type ATPase that functions as copper importer, maintain the copper level under low copper conditions through an increase in ctpB and a decrease in ctpV. However, when there is a rapid increase in copper levels, it has been observed that *ctpB*-knockout *M. tuberculosis*-infected mice exhibit increased resistance to copper, which enhances the efflux of copper. (Shey-Njila et al., 2022).

### 4.3 Cryptococcus

Notably, fungal pathogens such as *Cryptococcus* are able to regulate host copper levels through copper-dependent ATPases. In *Cryptococcus neoformans*-infected mice, bronchoalveolar lavage fluid is collected to measure copper-related proteins. The dominant cell type, alveolar macrophages, displayed a substantial decrease in ATP7A, which tends to be caused by the high Cu binding ability of metallothioneins. (Ding et al., 2013).

### 4.4 IAV

IAV, an enveloped virus from the *Orthomyxoviridae* family, is a notorious causative agent of recurrent seasonal respiratory disease. HA and NA, two N-glycoproteins protruding from the outer layer of the envelope, play essential roles in viral entry and egress. HA recognizes the N-acetylneuraminic sialic acid (NANA) residue on the cell surface to enter the host cell, while NA cleaves the sialic acid, facilitating virion excretion. Another outer layer protein, Matrix 2 (M2) tetramer, forms a proton channel to maintain a suitable pH for viral entry. (Vasin et al., 2014) IAV enters the host cell via endocytosis. Other proteins, including Matrix 1 (M1), viral nucleoprotein (NP), and non-structural proteins 1 and 2, are also involved in the lifecycle of IAV. (Samji, 2009).

The influence of copper levels on IAV replication and viral protein synthesis has been previously examined. Exogenous copper at a specific concentration can reduce the NA activity in H9N2, but it might not be mediated by the copper antiviral effect. (Horie et al., 2008) Copper regulators ATP7A and CTR1 were identified as potential factors affecting influenza virus replication in H1N1-infected human lung cells (A549). Rupp et al. conducted precise research on IAV replication features in *Atp7a* knockdown cells. (Rupp et al., 2017) The results demonstrated a mild decrease (1.4-fold) in virus production in *Atp7a* knockdown cells, with significant reductions in viral RNA and polymerase activity. Alongside viral RNA, the synthesis of NP and M1 proteins was also diminished in *Atp7a* knockdown cells. Furthermore, IAV appeared to disrupt the intracellular localization pattern of ATP7A, resulting in a dispersed vesicular pattern in the cytoplasm. (Rupp et al., 2017).

Apart from direct influences by the virus, ATP7A is involved in other interactions with IAV. IAV-induced increase of ROS is necessary for successful replication, and SOD1, a ROS scavenger, can be inhibited by IAV. The viral infection leads to the downregulation of specificity protein 1 (Sp1), a dominant cis-acting regulatory element of SOD1, which reduces the quantity and activity of SOD1. (Pyo et al., 2014) Knockdown of *atp7a* in zebrafish resulted in decreased *sod1* and *sp1* transcription, suggesting the expression of ATP7A positively regulates SOD1. (Chen et al., 2011) Therefore, disruption of ATP7A during IAV infection may impair SOD1 function, leading to copper-induced ROS accumulation and cellular autophagy. The ATP7A/autophagosome interaction is believed to enhance M2-triggered autophagy in a manner that increases ROS levels. (Wang et al., 2019; Puchkova et al., 2021).

### 4.5 ZIKV

ZIKV, a member of the *Flavivirida*e family, is an enveloped single-stranded RNA virus transmitted by various *Aedes* mosquitoes. The symptoms are mostly self-limiting, including fever, headache, arthralgia, myalgia, and maculopapular rash. However, newborns whose mothers are infected with ZIKV during pregnancy are reportedly born with a severe abnormality of the central nervous system (CNS). (Mlakar et al., 2016) The virus passes through the placental barrier, targeting various cells, including the central or peripheral nervous system, and then interacts with host factors, such as glycosaminoglycans and the C-type lectin DC-SIGN, to complete the infection. (Sirohi and Kuhn, 2017).

In the CNS, astrocytes maintain brain copper homeostasis. Copper-dependent ATPase, mostly ATP7A, controls astrocyte copper excretion. (Dringen et al., 2013) Copper disorder has been linked to some neurodegenerative features, including Alzheimer’s disease and Menke’s disease. Coincidentally, ZIKV infection demonstrates similar features to copper dysmetabolism. (Ledur et al., 2020; Witt et al., 2021) A recent study revealed that ATP7B expression was downregulated in astrocytes (induced pluripotent stem cells) after ZIKV infection. (Puig-Pijuan et al., 2022) Other copper transporters and chaperones had different trends: CTR1 saw a significant reduction overall; the gene of Atox1 was upregulated; and COMMD1, the regulator of ATP7A/B localization, was slightly upregulated. The copper accumulation mediated by a reduction in ATP7B might lead to oxidative stress in the host cell.

## 5 Conclusion and prospect

The copper metabolism of mammals largely depends on various transporters, including copper importer CTR1, copper exporter ATP7A/B, and copper chaperones, to maintain an intracellular and systemic balance. CRT1 is incorporated with cuprous ions in a high-affinity manner to transmit the kations into cells. ATP7A and ATP7B share some regulators and co-transporters, such as COMMD1 and Vps35. However, they have different distributions in tissues, resulting in slightly diverse functions in copper secretion. Copper homeostasis is crucial for the human body, especially the nervous system, and its dysmetabolism might be associated with neurodegeneration. (Scheiber et al., 2014; Wen et al., 2021).

Interestingly, microbial infection can affect copper metabolism in two aspects: animal hosts actively regulate copper levels in response to pathogen invasion; conversely, pathogens disturb cellular copper levels to cause lesions. As mentioned earlier, infection with *S. enterica* and *M. tuberculosis* leads to an upregulation of ATP7A. These bacteria employ copper pumps, such as CopA/GolT and ctpV/ctpB, to counteract the elevated copper levels. Other microbes, including *Cryptococcus neoformans*, IAV and ZIKV directly influence the regulation or localization of ATP7A/B. Recently, the endocytic recycling of angiotensin converting enzyme 2 (ACE2), glucose transporter type 1 (GLUT1), hydroxytryptamine receptor 4 (HTR4) and adrenoceptor beta 1 (ADRB1) was inhibited by SARS-CoV-2 S, possibly explaining some COVID symptoms associated with deficiency of those surface proteins. (Ren et al., 2022a; Ren et al., 2022b; Lv et al., 2023) The underlying mechanisms are that the spike (S) protein from SARS-CoV-2 binds to SNX27’s PDZ domain and reduces the association between SNX27 and Vps26A. (Ren et al., 2022a; Ren et al., 2022b) Since ATP7A is also a cargo of SNX27 in the recycling pathway, (Steinberg et al., 2013) SARS-CoV-2 S protein may suppress ATP7A recycling to the cell surface by associating with SNX27. That creates a ‘pseudo-ATP7A-deficiency’ pattern (**Figure 2B**).

Similar to SARS-CoV-2, some viruses target the protein sorting process of host cells and disrupt ion balance by influencing ion transporters. For instance, IAV targets ATP7A to disturb the host cellular copper balance, particularly causing loss of smell, (Doty et al., 2014) consistent with olfactory dysfunction associated with the systematic copper disorder. (Adamson et al., 2021) Interestingly, some bacteria use their prokaryotic copper transport ATPases, which share part of the structure with ATP7A/B, to maintain viability by exporting copper. For example, *S. Typhimurium* stimulates host copper-dependent ATPases expression and trafficking for host antibacterial effects. In turn, it utilizes procaryotic ATPases to relieve host copper threats. Additionally, there are bacteria and fungi which affect copper regulation excluding ATP7A/B. For instance, *Streptococcus pneumonia (S. pneumonia)*, using CopA to maintain copper levels, demonstrates high toxicity on the depletion of lung macrophages, which proves to be a main source of lung copper. (Johnson et al., 2015) And *Candida albicans* senses copper levels via two distinct copper regulators, Mac1 and Cup2, which can induce *CTR1* and the gene of MTs, to cope with host copper starvation or accumulation. (Besold et al., 2016) The interaction between microbes and host factors brings both chance and challenge in microbial infection treatment.

In conclusion, our review has focused on the characteristics and trafficking mechanisms of ATP7A/B, as well as the role of copper in antimicrobial responses, with a particular emphasis on the interplay between host copper-dependent ATPase and microbial infections. Additionally, we suggest that viral infections may disrupt the endocytic recycling process, leading to disturbances in copper metabolism. We believe that the link between microbial infections and copper metabolism can help elucidate specific clinical syndromes and provide new avenues for the prevention and treatment of infectious diseases.

## Author contributions

YZ: Writing – original draft. LZ: Conceptualization, Writing – review & editing.
